# Supplementary material for: The Systems Biology Research Tool: evolvable open-source software
Source: BMC Syst Biol. 2008 Jun 29;2:55. doi: 10.1186/1752-0509-2-55 (PMC2446383; doi:10.1186/1752-0509-2-55)
Supplement: Additional file 1 — SBRT Archive. An archive of the current version of the Systems Biology Research Tool. [file 1752-0509-2-55-S1.zip › sbrt-1.4.0/doc/developers_guide/api/sbrt/kernel/fba/class-use/Fluxome.html]

Uses of Interface sbrt.kernel.fba.Fluxome


|  |  |  |
| --- | --- | --- |
| **Overview** \| **Package** \| **Class** \| **Use** \| **Tree** \| **Deprecated** \| **Index** \| **Help** | |  |
| PREV   NEXT | **FRAMES**    **NO FRAMES**     **All Classes** |  |


---


## **Uses of Interface sbrt.kernel.fba.Fluxome**

| Packages that use Fluxome | |
| --- | --- |
| **sbrt.kernel.fba** | Provides classes and interfaces for creating objects relevant to Flux Balance Analysis. |
| **sbrt.kernel.fba.comp** | Provides classes and interfaces for performing Flux Balance Analysis computations. |
| **sbrt.kernel.fba.proc** | Provides classes and interfaces for executing Flux Balance Analysis processes. |
| **sbrt.shell.io.fba** | Provides classes and interfaces for reading and writing files relevant to Flux Balance Analysis. |
| **sbrt.shell.mng.fba** | Provides classes and interfaces for creating Flux Balance Analysis process managers. |
| **sbrt.shell.text.fba** | Provides classes and interfaces for defining formats for objects relevant to Flux Balance Analysis. |

Uses of Fluxome in sbrt.kernel.fba

| Subinterfaces of Fluxome in sbrt.kernel.fba | |
| --- | --- |
| `interface` | `CatalyzedFluxome`             This interface is used to represent fluxomes with catalyzed reactions. |
| `interface` | `IrrevRxnFluxome`             This class is used to represent fluxomes that contain only irreversible reactions. |

| Classes in sbrt.kernel.fba that implement Fluxome | |
| --- | --- |
| `class` | `IrrevRxnFluxomeV1`             This class is a concrete implementation of IrrevRxnFluxome. |

| Methods in sbrt.kernel.fba that return Fluxome | |
| --- | --- |
| `Fluxome` | `Fluxome.replaceConstraints(FluxConstraints constraints, double constraintTolerance)`             Returns a copy of this fluxome, but with all constraints replaced by those provided. |

Uses of Fluxome in sbrt.kernel.fba.comp

| Classes in sbrt.kernel.fba.comp with type parameters of type Fluxome | |
| --- | --- |
| `interface` | `FluxomeReducer<T extends Fluxome>`             This interface is used to represent algorithms that reduce the size of fluxomes. |

| Methods in sbrt.kernel.fba.comp that return Fluxome | |
| --- | --- |
| `Fluxome` | `InitialPointComputer.getFluxome()`             Returns the fluxome used by this computer. |
| `Fluxome` | `FluxCapComputer.getFluxome()`             Returns the fluxome for which flux caps are computed. |
| `Fluxome` | `FluxCvComputer.getFluxome()`             Returns the fluxome used to construct this flux constraint-variation computer. |

| Methods in sbrt.kernel.fba.comp with parameters of type Fluxome | |
| --- | --- |
| `static LinearSystemSolution<java.lang.String>` | `FbaSolver.solve(Fluxome fluxome, LinearSystemSolver<java.lang.String> solver)`             Solves *Sv = 0*, where *S* is the stoichiometry matrix of the provided fluxome and *v* is the vector of fluxes.** |

Constructors in sbrt.kernel.fba.comp with parameters of type Fluxome

`FluxCapComputer(Fluxome fluxome,
java.util.Collection<LinearComb<java.lang.String>> allCycles)`
  
          Constructs a new flux cap computer from the cycles contained
in the provided fluxome.

`FluxCapComputer(Fluxome fluxome,
DoubleMatrix<?,java.lang.String> cycleMatrix)`
  
          Constructs a new flux cap computer from the cycles contained
in the provided fluxome.

Uses of Fluxome in sbrt.kernel.fba.proc

| Classes in sbrt.kernel.fba.proc with type parameters of type Fluxome | |
| --- | --- |
| `class` | `FluxomeReducerProcess<T extends Fluxome>`             This class is used to execute fluxome reduction processes. |

| Methods in sbrt.kernel.fba.proc that return Fluxome | |
| --- | --- |
| `Fluxome` | `CvFbaOptimizationExperiment.getFluxome()`             Returns the fluxome used in this experiment. |
| `Fluxome` | `FluxCapProcess.getFluxome()`             Returns the fluxome for which flux caps are computed. |
| `Fluxome` | `CdHarFluxVectorProcess.getFluxome()`             Returns the fluxome for which flux vectors are to be generated. |
| `Fluxome` | `ObjectiveFunctionAnalysis.getFluxome()`             Returns the fluxome used in this experiment. |
| `Fluxome` | `RandomObjectiveGenerator.getFluxome()`             Returns the fluxome for which random objective functions are to be generated. |
| `Fluxome` | `FluxIntervalProcess.getFluxome()`             Returns the fluxome used in this experiment. |
| `Fluxome` | `InitialPointGenerator.getFluxome()`             Returns the fluxome used in this initial point experiment. |
| `Fluxome` | `RxnDeletionProcess.getFluxome()`             Returns the fluxome used by the reaction deletion computer. |
| `Fluxome` | `CvRxnDeletionProcess.getFluxome()`             Returns the fluxome used by the reaction deletion computer. |
| `Fluxome` | `StoichVariationProcess.getFluxome()`             Returns the fluxome used in this process. |
| `Fluxome` | `FluxActivityProcess.getFluxome()`             Returns the fluxome used in this flux activity experiment. |
| `Fluxome` | `SvInitialPointProcess.getFluxome()`             Returns the fluxome used in this initial point experiment. |
| `Fluxome` | `CvObjectiveFunctionProcess.getFluxome()`             Returns the fluxome used in this experiment. |
| `abstract  Fluxome` | `FbaProcess.getFluxome()`             Returns the fluxome used in this experiment. |
| `Fluxome` | `FbaOptimizationProcess.getFluxome()`             Returns the fluxome used in this experiment. |
| `Fluxome` | `CvFluxIntervalProcess.getFluxome()`             Returns the fluxome used in this experiment. |
| `Fluxome` | `SvCdHarFluxVectorProcess.getFluxome()`             Returns the fluxome for which flux vectors are to be generated. |
| `Fluxome` | `FluxPlasticityProcess.getFluxome()`             Returns the fluxome used in this flux plasticity experiment. |
| `Fluxome` | `FbaSolverProcess.getFluxome()`             Returns the fluxome for which Sv = 0 is solved. |

Constructors in sbrt.kernel.fba.proc with parameters of type Fluxome

`FbaSolverProcess(Fluxome fluxome)`
  
          Constructs a new FBA solver process.

`FluxActivityProcess(Fluxome fluxome)`
  
          Constructs a new flux activity experiment.

`FluxPlasticityProcess(Fluxome fluxome)`
  
          Constructs a new flux plasticity experiment.

`RandomObjectiveGenerator(Fluxome fluxome)`
  
          Constructs a new random objective function generator.

Uses of Fluxome in sbrt.shell.io.fba

| Methods in sbrt.shell.io.fba that return Fluxome | |
| --- | --- |
| `Fluxome` | `FluxCapInputFile.getFluxome()`             Returns the fluxome used to verify reaction names. |

Uses of Fluxome in sbrt.shell.mng.fba

| Methods in sbrt.shell.mng.fba with parameters of type Fluxome | |
| --- | --- |
| `static java.util.Map<java.lang.String,Or<And<java.lang.String>>>` | `FbaManager.getCatalysts(Fluxome fluxome, java.lang.String fileName)`             Returns the reaction-catalyst associations contained in the file with the provided name. |
| `static java.util.Map<java.lang.String,Or<And<java.lang.String>>>` | `FbaManager.getCatalysts(Fluxome fluxome, java.lang.String fileName, java.io.PrintWriter out)`             Returns the reaction-catalyst associations contained in the file with the provided name. |
| `static FluxConstraints` | `FbaOptManager.getConstraints(ManagerInput<java.lang.String,java.lang.String> input, java.io.PrintWriter out, Fluxome fluxome)`             Opens the file denoted by `FbaOptManager.CONSTRAINTS_FILE` in the provided manager input and returns the set of flux constraints it contains. |
| `static FluxConstraints` | `RandomFluxVectorManager.getConstraints(ManagerInput<java.lang.String,java.lang.String> input, java.io.PrintWriter out, Fluxome fluxome)`             Opens the file denoted by `FbaOptManager.CONSTRAINTS_FILE` in the provided manager input and returns the set of flux constraints it contains. |
| `static FluxConstraints` | `FbaOptManager.getConstraints(java.lang.String fileName, Fluxome fluxome)`             Returns the set of constraints contained in the file with the provided name. |
| `static FluxConstraints` | `RandomFluxVectorManager.getConstraints(java.lang.String fileName, Fluxome fluxome)`             Returns the set of constraints contained in the file with the provided name. |
| `static MapOutputFile<java.lang.String,java.lang.Double>` | `InitialPointManager.getCVectorFile(ManagerInput<java.lang.String,java.lang.String> input, java.io.PrintWriter out, Fluxome fluxome)`             Returns an empty output file that is suitable for storing a single C vector with the name denoted by `InitialPointManager.P_VECTOR_FILE_NAME` in the provided input. |
| `static FluxIntervalInputFile` | `IntervalVariationManager.getCvInputFile(ManagerInput<java.lang.String,java.lang.String> input, java.io.PrintWriter out, Fluxome fluxome)`             Returns the constraint variation input with the name denoted by `IntervalVariationManager.CV_FILE` in the provided manager input. |
| `static FluxIntervalInputFile` | `IntervalVariationManager.getCvInputFile(java.lang.String fileName, Fluxome fluxome)`             Returns the constraint variation input file with the provided name. |
| `static java.util.List<LinearComb<java.lang.String>>` | `FluxCapManager.getCycles(ManagerInput<java.lang.String,java.lang.String> input, java.io.PrintWriter out, Fluxome fluxome)`             Opens the file denoted by `FluxCapManager.CYCLE_FILE` in the provided manager input and returns the list of cycles it contains. |
| `static java.util.Set<java.lang.String>` | `FbaOptManager.getDataHeaders(ManagerInput<java.lang.String,java.lang.String> input, Fluxome fluxome)`             Parses and returns the value for `AbstractProcessManager.DATA_HEADERS` in the provided manager input. |
| `static java.util.LinkedHashSet<java.lang.String>` | `FbaOptManager.getDataHeaders(java.lang.String dataHeaders, Fluxome fluxome)`             Parses the provided data header string and returns the corresponding set. |
| `static UnmodifiableMap<java.lang.String,java.util.Set<LinearComb<java.lang.String>>>` | `MsFluxIntervalManager.getFluxCaps(ManagerInput<java.lang.String,java.lang.String> input, java.io.PrintWriter out, Fluxome fluxome)`             Returns the flux caps contained in the file with the name specified by `MsFluxIntervalManager.FLUX_CAP_FILE` in the provided input. |
| `static MapInputFile<java.lang.String,Interval>` | `FluxPlasticityManager.getFluxIntervalFile(ManagerInput<java.lang.String,java.lang.String> input, java.io.PrintWriter out, Fluxome fluxome)`             Returns the flux interval input with the name denoted by `FluxPlasticityManager.FLUX_INTERVAL_FILE` in the provided manager input. |
| `static MapInputFile<java.lang.String,Interval>` | `FluxPlasticityManager.getFluxIntervalFile(java.lang.String fileName, Fluxome fluxome)`             Returns the flux interval input file with the provided name. |
| `static LinearSystemSolution<java.lang.String>` | `FbaManager.getFluxomeSolution(ManagerInput<java.lang.String,java.lang.String> input, java.io.PrintWriter out, Fluxome fluxome)`             Opens the file denoted by `FbaManager.SYSTEM_SOLUTION_FILE` in the provided manager input and returns the linear system solution it contains. |
| `static java.util.Map<java.lang.String,java.lang.Double>` | `CdHarFluxVectorManager.getFluxVector(ManagerInput<java.lang.String,java.lang.String> input, java.io.PrintWriter out, Fluxome fluxome, double tolerance)`             Opens the file denoted by `CdHarManager.INITIAL_POINT_FILE` in the provided manager input and returns the flux vector it contains. |
| `static java.util.Map<java.lang.String,java.lang.Double>` | `RandomFluxVectorManager.getFluxVector(ManagerInput<java.lang.String,java.lang.String> input, java.io.PrintWriter out, Fluxome fluxome, double tolerance)`             Opens the file denoted by `FbaManager.FLUX_VECTOR_FILE` in the provided manager input and returns the flux vector it contains. |
| `static java.util.Map<java.lang.String,java.lang.Double>` | `RandomFluxVectorManager.getFluxVector(java.lang.String fileName, Fluxome fluxome, double tolerance)`             Returns the flux vector contained in the file with the provided name. |
| `static MapOutputFile<java.lang.String,java.lang.Double>` | `SvInitialPointManager.getFluxVectorFile(ManagerInput<java.lang.String,java.lang.String> input, java.io.PrintWriter out, Fluxome fluxome)`             Returns an empty output file that is suitable for storing multiple flux vectors. |
| `static MapOutputFile<java.lang.String,java.lang.Double>` | `InitialPointManager.getFluxVectorFile(ManagerInput<java.lang.String,java.lang.String> input, java.io.PrintWriter out, Fluxome fluxome)`             Returns an empty output file that is suitable for storing a single flux vector. |
| `static MapInputFile<java.lang.String,java.lang.Double>` | `FluxActivityManager.getFluxVectorFile(ManagerInput<java.lang.String,java.lang.String> input, java.io.PrintWriter out, Fluxome fluxome)`             Returns the flux vector input file specified by the provided input. |
| `static MapInputFile<java.lang.String,java.lang.Double>` | `FluxActivityManager.getFluxVectorFile(java.lang.String fileName, FileFormat format, Fluxome fluxome)`             Returns the flux vector input file with the provided name. |
| `static InputFile<java.util.Map<java.lang.String,java.lang.Double>>` | `SvCdHarFluxVectorManager.getInitialFluxVectorFile(ManagerInput<java.lang.String,java.lang.String> input, java.io.PrintWriter out, Fluxome fluxome)`             Returns the input file containing a collection of initial flux vectors. |
| `static InputFile<java.util.Set<java.lang.String>>` | `RxnDeletionManager.getInputFile(ManagerInput<java.lang.String,java.lang.String> input, java.io.PrintWriter out, Fluxome fluxome)`             Parses and returns the value for `RxnDeletionManager.RXN_DELETION_FILE` in the provided manager input. |
| `static TextInputFile<LinearComb<java.lang.String>>` | `ObjectiveFunctionManager.getInputFile(ManagerInput<java.lang.String,java.lang.String> input, java.io.PrintWriter out, Fluxome fluxome)`             Returns the file denoted by `ObjectiveFunctionManager.OBJ_FUNCT_FILE` in the provided manager input. |
| `static TextInputFile<LinearComb<java.lang.String>>` | `ObjectiveFunctionManager.getInputFile(java.lang.String fileName, FileFormat format, Fluxome fluxome)`             Returns the input file with the provided name. |
| `static InputFile<java.util.Set<java.lang.String>>` | `RxnDeletionManager.getInputFile(java.lang.String fileName, Fluxome fluxome)`             Returns the reaction name input file with the provided name. |
| `static TextInputFile<LinearComb<java.lang.String>>` | `ObjectiveFunctionManager.getInputFile(java.lang.String fileName, Fluxome fluxome)`             Returns the input file with the provided name. |
| `static FluxIntervalInputFile` | `IntervalVariationManager.getIntervalInputFile(java.lang.String fileName, Fluxome fluxome)`             Returns the flux interval input file with the provided name. |
| `static FluxIntervalInputFile` | `IntervalVariationManager.getIntervalInputFile(java.lang.String fileName, Fluxome fluxome, MapFormat<LinearComb<java.lang.String>,Interval> mapFormat)`             Returns the flux interval input file with the provided name. |
| `static FluxConstraints` | `InitialPointManager.getIntervals(ManagerInput<java.lang.String,java.lang.String> input, java.io.PrintWriter out, Fluxome fluxome)`             Opens the file denoted by `InitialPointManager.FLUX_INTERVAL_FILE` in the provided manager input and returns the set of flux intervals it contains. |
| `static FluxConstraints` | `InitialPointManager.getIntervals(java.lang.String fileName, Fluxome fluxome)`             Returns the set of flux intervals contained in the file with the provided name. |
| `static MultiOutputFiles<ComoFile<java.lang.String,java.lang.Double>,java.util.Map<? extends java.lang.String,? extends java.lang.Double>>` | `SvCdHarFluxVectorManager.getMulitComoFiles(ManagerInput<java.lang.String,java.lang.String> input, java.io.PrintWriter out, java.util.Iterator<java.lang.String> fileNameSource, Fluxome fluxome)`             Returns a source of output files. |
| `static MultiOutputFiles<ColumnOrFbaOptOutputFile,FbaOptComputer.Results>` | `FbaOptManager.getMultiColOrOutputFiles(ManagerInput<java.lang.String,java.lang.String> input, java.io.PrintWriter out, java.util.Iterator<java.lang.String> fileNameSource, java.lang.String fileNameSourceName, Fluxome fluxome)`             Please consult the source code for a complete description of this method's actions. |
| `static MultiOutputFiles<? extends FbaOptOutputFile,FbaOptComputer.Results>` | `FbaOptManager.getMultiFileOutput(ManagerInput<java.lang.String,java.lang.String> input, java.io.PrintWriter out, java.util.Iterator<java.lang.String> fileNameSource, java.lang.String fileNameSourceName, Fluxome fluxome)`             Please consult the source code for a complete description of this method's actions. |
| `static MapOutputFile<java.lang.String,java.lang.Double>` | `CdHarFluxVectorManager.getMultiFluxVectorOutputFile(ManagerInput<java.lang.String,java.lang.String> input, java.io.PrintWriter out, Fluxome fluxome)`             Returns an empty output file that is suitable for storing multiple flux vectors. |
| `static MapOutputFile<java.lang.String,java.lang.Double>` | `FbaManager.getMultiFluxVectorOutputFile(ManagerInput<java.lang.String,java.lang.String> input, java.io.PrintWriter out, Fluxome fluxome)`             Returns an empty output file that is suitable for storing multiple flux vectors. |
| `static MapOutputFile<java.lang.String,java.lang.Double>` | `FbaManager.getMultiFluxVectorOutputFile(java.lang.String fileName, FileFormat fileFormat, java.util.Set<java.lang.String> rxnNameHeaders, Fluxome fluxome)`             Returns an empty output file that is suitable for storing multiple flux vectors. |
| `static ComoFile<java.lang.String,Interval>` | `SimpleFluxIntervalManager.getMultiResultsOutputFile(ManagerInput<java.lang.String,java.lang.String> input, java.io.PrintWriter out, Fluxome fluxome)`             Returns an empty output file that is suitable for storing multiple sets of flux interval results. |
| `static FbaOptOutputFile` | `FbaOptManager.getMultiResultsOutputFile(ManagerInput<java.lang.String,java.lang.String> input, java.io.PrintWriter out, Fluxome fluxome)`             Returns an empty output file that is suitable for storing multiple FBA optimization results. |
| `static FbaOptOutputFile` | `FbaOptManager.getMultiResultsOutputFile(java.lang.String fileName, FileFormat fileFormat, java.util.Set<java.lang.String> dataHeaders, Fluxome fluxome)`             Returns an empty output file that is suitable for storing multiple FBA optimization results. |
| `static ComoFile<java.lang.String,Interval>` | `SimpleFluxIntervalManager.getMultiResultsOutputFile(java.lang.String fileName, java.util.Set<java.lang.String> headers, Fluxome fluxome)`             Returns an empty output file that is suitable for storing multiple sets of flux interval results. |
| `static MultiOutputFiles<RowOrFbaOptOutputFile,FbaOptComputer.Results>` | `FbaOptManager.getMultiRowOrOutputFiles(ManagerInput<java.lang.String,java.lang.String> input, java.io.PrintWriter out, java.util.Iterator<java.lang.String> fileNameSource, Fluxome fluxome)`             Please consult the source code for a complete description of this method's actions. |
| `static MultiOutputFiles<SerFbaOptOutputFile,FbaOptComputer.Results>` | `FbaOptManager.getMultiSerOutputFiles(ManagerInput<java.lang.String,java.lang.String> input, java.io.PrintWriter out, java.util.Iterator<java.lang.String> fileNameSource, java.lang.String fileNameSourceName, Fluxome fluxome)`             Please consult the source code for a complete description of this method's actions. |
| `static LinearComb<java.lang.String>` | `FbaOptManager.getObjectiveFunction(ManagerInput<java.lang.String,java.lang.String> input, java.io.PrintWriter out, Fluxome fluxome)`             Parses and returns the value for `FbaOptManager.OBJECTIVE_FUNCTION` in the provided manager input. |
| `static ComoFile<java.lang.String,java.lang.Double>` | `ExtremeCurrentManager.getOutputFile(ManagerInput<java.lang.String,java.lang.String> input, java.io.PrintWriter out, Fluxome fluxome, java.util.Set<java.lang.String> rxnNames)`             Returns an output file to which extreme currents can be written. |
| `static ComoFile<java.lang.String,java.lang.Double>` | `ExtremeCurrentManager.getOutputFile(java.lang.String fileName, java.io.PrintWriter out, Fluxome fluxome, java.util.Set<java.lang.String> rxnNames)`             Returns an output file to which extreme currents can be written. |
| `static RxnNameExprFormat<LinearComb<java.lang.String>,java.lang.String>` | `FbaManager.getRxnNameExprFormat(Fluxome f)`             Returns the default reaction name expression format. |
| `static java.util.Set<java.lang.String>` | `FbaManager.getRxnNameHeaders(ManagerInput<java.lang.String,java.lang.String> input, Fluxome fluxome)`             Parses and returns the value for `AbstractProcessManager.DATA_HEADERS` in the provided manager input. |
| `static java.util.LinkedHashSet<java.lang.String>` | `FbaManager.getRxnNameHeaders(java.lang.String dataHeaders, Fluxome fluxome)`             Parses and returns the set of reaction names contained in the provided string. |
| `static MapOutputFile<java.lang.String,java.lang.Double>` | `FbaManager.getSingleFluxVectorOutputFile(ManagerInput<java.lang.String,java.lang.String> input, java.io.PrintWriter out, Fluxome fluxome)`             Returns an empty output file that is suitable for storing a single flux vector. |
| `static MapOutputFile<java.lang.String,java.lang.Double>` | `FbaManager.getSingleFluxVectorOutputFile(java.lang.String fileName, FileFormat fileFormat, java.util.Set<java.lang.String> rxnNameHeaders, Fluxome fluxome)`             Returns an empty output file that is suitable for storing a single flux vector. |
| `static RomoFile<java.lang.String,Interval>` | `SimpleFluxIntervalManager.getSingleResultOutputFile(ManagerInput<java.lang.String,java.lang.String> input, java.io.PrintWriter out, Fluxome fluxome)`             Returns an empty output file that is suitable for storing a single set of flux interval results. |
| `static FbaOptOutputFile` | `FbaOptManager.getSingleResultOutputFile(ManagerInput<java.lang.String,java.lang.String> input, java.io.PrintWriter out, Fluxome fluxome)`             Returns an empty output file that is suitable for storing a single FBA optimization result. |
| `static FbaOptOutputFile` | `FbaOptManager.getSingleResultOutputFile(java.lang.String fileName, FileFormat fileFormat, java.util.Set<java.lang.String> dataHeaders, Fluxome fluxome)`             Returns an empty output file that is suitable for storing a single FBA optimization result. |
| `static RomoFile<java.lang.String,Interval>` | `SimpleFluxIntervalManager.getSingleResultOutputFile(java.lang.String fileName, java.util.Set<java.lang.String> headers, Fluxome fluxome)`             Returns an empty output file that is suitable for storing a single set of flux interval results. |
| `static FluxIntervalInputFile` | `IntervalVariationManager.getSvInputFile(ManagerInput<java.lang.String,java.lang.String> input, java.io.PrintWriter out, Fluxome fluxome)`             Returns the space variation input with the name denoted by `IntervalVariationManager.FLUX_INTERVAL_FILE` in the provided manager input. |
| `static void` | `NetworkInfoManager.printBasicInfo(Fluxome fluxome, java.io.PrintWriter out)`             Prints basic information about the provided fluxome. |
| `static void` | `FbaManager.printFluxomeInfo(Fluxome fluxome, java.io.PrintWriter out)`             Prints basic information about the provided fluxome. |

Uses of Fluxome in sbrt.shell.text.fba

| Methods in sbrt.shell.text.fba that return Fluxome | |
| --- | --- |
| `Fluxome` | `FluxCapFormatV1.getFluxome()`             Returns the fluxome used to verify reaction names. |
| `Fluxome` | `CatalystSetFormat.getFluxome()`             Returns the fluxome used to verify enzyme names. |
| `Fluxome` | `ConstraintsFileLineFormatV1.getFluxome()`             Returns the fluxome used to verify reaction names. |
| `Fluxome` | `FluxVectorFormatV1.getFluxome()`             Returns the fluxome used to verify reaction names. |
| `Fluxome` | `RxnNameExprFormat.getFluxome()`             Returns the fluxome used to verify reaction names. |
| `Fluxome` | `ChemSpeciesVerifier.getFluxome()`             Returns the fluxome used to create this chemical species verifier. |
| `Fluxome` | `RxnNameExprFormatV1.getFluxome()`             Returns the fluxome used to verify reaction names. |
| `Fluxome` | `FluxVectorFormat.getFluxome()`             Returns the fluxome used to verify reaction names. |
| `Fluxome` | `RxnNameOrExprFormatV1.getFluxome()`             Returns the fluxome used to verify reaction names. |
| `Fluxome` | `FbaOptHeaderParserV1.getFluxome()`             Returns the fluxome used to verify reaction names. |
| `Fluxome` | `RxnNameOrExprFormat.getFluxome()`             Returns the fluxome used to verify reaction names. |
| `Fluxome` | `FbaOptHeaderFormatter.getFluxome()`             Returns the fluxome used to verify reaction names. |
| `Fluxome` | `RxnNameSetFormat.getFluxome()`             Returns the fluxome used to verify reaction names. |
| `Fluxome` | `ConstraintsFileLineFormatV2.getFluxome()`             Returns the fluxome used to verify reaction names. |
| `Fluxome` | `RxnNameListFormatV1.getFluxome()`             Returns the fluxome used to verify reaction names. |
| `Fluxome` | `FbaOptHeaderFormatterV1.getFluxome()`             Returns the fluxome used to verify reaction names. |
| `Fluxome` | `FbaOptHeaderParser.getFluxome()`             Returns the fluxome used to verify reaction names. |
| `Fluxome` | `FluxomeSolFileLineFormatV1.getFluxome()`             Returns the fluxome used to verify reaction names. |
| `Fluxome` | `RxnNameVerifier.getFluxome()`             Returns the fluxome used to create this reaction name checker. |
| `Fluxome` | `RxnNameSetFormatV1.getFluxome()`             Returns the fluxome used to verify reaction names. |
| `Fluxome` | `RxnNameListFormat.getFluxome()`             Returns the fluxome used to verify reaction names. |
| `Fluxome` | `FluxomeSolutionFileLineFormat.getFluxome()`             Returns the fluxome used to verify reaction names. |
| `Fluxome` | `FluxCapFormat.getFluxome()`             Returns the fluxome used to verify reaction names. |
| `Fluxome` | `FbaOptFileLineFormatV1.getFluxome()`             Returns the fluxome used to verify reaction names. |
| `Fluxome` | `ConstraintsFileLineFormat.getFluxome()`             Returns the fluxome used to verify reaction names. |
| `Fluxome` | `FbaOptFileLineFormat.getFluxome()`             Returns the fluxome used to verify reaction names. |

Constructors in sbrt.shell.text.fba with parameters of type Fluxome

`ChemSpeciesVerifier(Fluxome fluxome)`
  
          Constructs a new chemical species verifier for the
provided fluxome.

`ConstraintsFileLineFormatV1(Fluxome fluxome)`
  
          Constructs a new contraints file line format.

`ConstraintsFileLineFormatV2(Fluxome fluxome)`
  
          Constructs a new contraints file line format.

`FbaOptFileLineFormatV1(Fluxome fluxome)`
  
          Constructs a new FBA optimization file line format.

`FbaOptHeaderFormatterV1(Fluxome fluxome)`
  
          Constructs a new FBA optimization header formatter.

`FbaOptHeaderParserV1(Fluxome fluxome)`
  
          Constructs a new FBA optimization header parser.

`FluxCapFormatV1(Fluxome fluxome)`
  
          Constructs a new flux cap file line format using the
provided fluxome.

`FluxomeSolFileLineFormatV1(Fluxome fluxome)`
  
          Constructs a new fluxome solution file line format.

`FluxVectorFormatV1(Fluxome fluxome)`
  
          Constructs a new flux vector format.

`RxnNameExprFormatV1(Fluxome fluxome)`
  
          Constructs a new reaction name expression format
using the provided fluxome.

`RxnNameListFormatV1(Fluxome fluxome)`
  
          Constructs a new reaction name list format
using the provided fluxome.

`RxnNameOrExprFormatV1(Fluxome fluxome)`
  
          Constructs a new reaction name and expression format.

`RxnNameSetFormatV1(Fluxome fluxome)`
  
          Constructs a new reaction name collection format
using the provided fluxome.

`RxnNameVerifier(Fluxome fluxome)`
  
          Constructs a new reaction name checker for the
provided fluxome.

---


|  |  |  |
| --- | --- | --- |
| **Overview** \| **Package** \| **Class** \| **Use** \| **Tree** \| **Deprecated** \| **Index** \| **Help** | |  |
| PREV   NEXT | **FRAMES**    **NO FRAMES**     **All Classes** |  |


---
